# Deletion of natriuretic peptide receptor C alleviates adipose tissue inflammation in hypercholesterolemic Apolipoprotein E knockout mice

**DOI:** 10.1111/jcmm.16931

**Published:** 2021-09-15

**Authors:** Cheng Cheng, Fei Xue, Wenhai Sui, Linlin Meng, Lin Xie, Cheng Zhang, Jianmin Yang, Yun Zhang

**Affiliations:** ^1^ Department of Cardiology The Key Laboratory of Cardiovascular Remodeling and Function Research Chinese Ministry of Education Chinese National Health Commission and Chinese Academy of Medical Sciences The State and Shandong Province Joint Key Laboratory of Translational Cardiovascular Medicine Qilu Hospital Cheeloo College of Medicine Shandong University Jinan China

**Keywords:** adipose tissue, atherosclerosis, inflammation, natriuretic peptide receptor C

## Abstract

The inflammation of adipose tissue is one of the most common secondary pathological changes in atherosclerosis, which in turn influences the process of atherosclerosis. Natriuretic peptides have been revealed important effect in regulating adipose metabolism. However, the relationship between natriuretic peptide receptor C and inflammation of adipose tissue in atherosclerosis remains unknown. This study aims to explore the effect natriuretic peptide receptor C exerts on the regulation of the adipose inflammation in atherosclerotic mice induced by western‐type diet and its overlying mechanisms. To clarify the importance of NPRC of adipose inflammation in atherosclerotic mice, NPRC expression was measured in mice fed with chow diet and western‐type diet for 12 weeks and we found a considerable increase in adipose tissue of atherosclerotic mice. Global NPRC knockout in mice was bred onto ApoE^−/−^ mice to generate NPRC^−/−^ApoE^−/−^ mice, which displayed remarked increase in browning of white adipose tissue and lipolysis of adipose tissue and decrease in adipose inflammation manifested by decreased macrophage invasion to form less CLS (crown‐like structure), reduced oxidative stress and alleviated expression of TNFα, IL‐6, IL‐1β and MCP1, but increased expression of adiponectin in adipose tissue. Moreover, our study showed that white adipose tissue browning in NPRC^−/−^ApoE^−/−^ atherosclerotic mice was associated with decreased inflammatory response through cAMP/PKA signalling activation. These results identify NPRC as a novel regulator for adipose inflammation in atherosclerotic mice by modulating white adipose tissue browning.

## 1 INTRODUCTION

Atherosclerosis, as a chronic inflammation‐related disease, characterized by endothelial dysfunction, inflammatory cell recruitment, lipid oxidation and form cell formation.^1^ Obesity is a major risk factor for atherosclerosis.^2^ Adipose tissue exerts its effect on energy metabolism, storing and releasing lipids, which is showed to be closely associated with the process of sorts of cardiovascular disease such as atherosclerosis, hypertension and coronary artery disease.^3^, ^4^, ^5^ In diet‐induced obesity, adipose tissue was characterized as inflamed and dysfunctional, which released more pro‐inflammatory adipocytokines and free fatty acids resulted in vascular inflammation and oxidative stress to enhance atherosclerosis in a systemic manner.^6^, ^7^ More insight into the relationship between adipose tissue and atherosclerosis may provide new knowledge and measures to protect patients with high mass of fat from the risk of atherosclerosis.

Adipose tissue is found throughout the body, which is mainly composed of adipocytes. Adipocytes can be approximately classified into three phenotypes (white, beige and brown) according to their different natures. Moreover, the phenotypes of adipocytes are not set in stone and they can transform to each other in distinct condition. White adipose tissue (WAT) is the most abundant adipose tissue type.^8^ In pathological condition, besides releasing fatty acids, white adipocytes can secrete lots of pro‐inflammatory factors, which recruit several types of immune cells such as macrophages into adipose tissue.^9^, ^10^ As a consequence, white adipose tissue gradually leads to systemic inflammation, which may play a role in the progress of atherosclerosis.^11^ In contrast to WAT, brown adipose tissue (BAT) is reported to alleviate hypertriglyceridemia^12^ and protect from atherosclerosis development in mice.^13^

Natriuretic peptides were found to regulate multiple physiological process including diuresis, natriuresis, vasorelaxant effects and vascular remodelling,^14^ which include atrial and brain natriuretic peptides (ANP and BNP) produced by cardiac cells and C‐type natriuretic peptide (CNP) released by endothelium.^15^ Besides, three main receptors for natriuretic peptides have been found, which classified into natriuretic peptide receptor A (NPRA), natriuretic peptide receptor B (NPRB), natriuretic peptide receptor C (NPRC).^16^ NPRC, regarded as just a clearance receptor for ANP, BNP and CNP a few years ago, has been received more and more attention from researchers because of its undetected function, such as the activity of inhibitory guanine nucleotide regulatory protein (Gi).^17^ There was a study revealing adipose specific deletion of NPRC enhanced the browning of white adipose tissue and protected against diet‐induced obesity and insulin resistance.^18^ Moreover, CNP was showed associated with maintaining vascular homeostasis.^19^ However, few researchers reported that the role of NPRC played in adipose inflammation accompanied with atherosclerosis.

Therefore, to clarify the effect NPRC exerts on adipose inflammation accompanied with atherosclerosis, we generate NPRC^−/−^ApoE^−/−^ mice to construct atherosclerotic models to explore adipose phenotype, metabolism and inflammation compared with ApoE^−/−^ mice, which not only uncover the roles NPRC plays in adipose inflammation, but also may provide new views for preventing atherosclerosis.

## 2 METHODS AND MATERIALS

### 2.1 Animal protocols

All animal experimental protocols were permitted by the Ethics Committee and the Scientific Investigation Board of Shandong University Qilu Hospital (Jinan, Shandong Province, China) according to the Animal Management Rules of the Chinese Ministry of Health. 8‐week‐old apolipoprotein E‐knockout (APOE^−/−^) male mice were bought from Beijing Viewsolid Biotechnology Co. LTD (Beijing, China). The NPRC^−/−^ mice were constructed using CRISPR technology from Beijing Viewsolid Biotechnology Co. LTD (Beijing, China) on the C57BL/6 background. The genotyping of the NPRC^−/−^ mice was confirmed through sequencing of the PCR fragments in the CRISPR‐targeting region amplified from genomic DNA isolated from tail tips with the following primers: Forward 5′‐TTGGCGAGTTACTGAAGG‐3′ and Reverse 5′‐CGGTCCACAAGACTGAAG‐3′. Then we cross the two kinds of mice to get NPRC^−/−^APOE^−/−^ mice. All animals were kept on a 12‐h light/12‐h dark cycle at 22℃ room temperature. The experimental animals were assigned to two groups (*n* = 25 per group): APOE^−/−^ group and APOE^−/−^NPRC^−/−^ group. All animals were fed with a 12‐h/12‐h light‐dark cycle at 22℃ room temperature with a high‐fat, high‐cholesterol Western‐type diet (WTD) (typically 0.2% cholesterol, 21% milk fat) and water freely available for 12 weeks. Different groups were collected in a randomized manner, and investigators were blinded to the allocation of different groups when doing surgeries and doing outcome evaluations.

### 2.2 Tissue preparation and histopathological analysis

For morphological studies, mice were euthanized with an overdose of pentobarbital sodium and perfused immediately with 4% paraformaldehyde in 0.1 M phosphate buffer, PH 7.4, for 5 min. BAT and WAT depots were dissected and further fixed by immersion in 4% paraformaldehyde overnight at 4℃. After a thorough rinse in phosphate buffer, tissue depots were dehydrated in ethanol, cleared in xylene and embedded in paraffin. Adipocyte size was measured on haematoxylin and eosin staining, and we quantified average adipocyte size in 5 serial sections (60 μm between sections). The quantification of adipocytes size was performed by researchers who were blinded to group allocation.

### 2.3 Reagents and antibodies

Detailed information for antibodies was listed in Table S1.

### 2.4 Histology examination and immunohistochemistry

Sections of adipose tissue from mice were dewaxed and then react with 0.3% H_2_O_2_ to block endogenous peroxidase, rinsed in PBS and incubated in 5% normal serum blocking solution. Sections were then incubated with primary antibodies overnight at 4℃. At the same time, we set up IgG negative control of the same species as the primary antibody and the solvent PBS control to verify the antibody and exclude false‐positive results. After a thorough rinse in PBS, sections were incubated in 1:200 v/v goat anti‐rabbit or goat anti‐mouse IgG biotinylated HRP‐conjugated secondary antibody solution in PBS for 30 min. DAB kit (ZSGB‐Bio, Beijing) and AEC kit (Solarbio, Beijing) were used for colour development. Haematoxylin was used to counterstain nuclei in immunohistochemical staining.

### 2.5 Apoptosis assay

Apoptosis was detected by TUNEL staining with an *in situ* cell death detection kit (Roche). First, sections of adipose tissue from mice were dewaxed. Then, sections were incubated in PBS with 0.1% Triton X‐100. Following the staining with TUNEL reaction mixture for 1h at 37℃. Finally, the sections were counterstained with DAPI and photographed via laser scanning confocal microscopy (LSM 710, Zeiss).

### 2.6 Reactive oxidative species assay

Reactive oxidative species were detected by DHE staining. First, sections of adipose tissue from mice were dewaxed. Then, sections were incubated in PBS with 0.1% Triton X‐100. Following the staining with DHE reaction solution for 1h at 37℃. Finally, the sections were photographed via laser scanning confocal microscopy (LSM 710, Zeiss).

### 2.7 RT‐PCR assay

Total RNA was extracted from isolated adipose tissue by using the RNeasy mini kit (Qiagen, 74106, Germany), which was reversed‐transcribed with a PrimeScript RT reagent kit with gDNA Eraser (TaKaRa, Japan) according to the manufacturer's instructions. Reverse‐transcription products of different samples were amplified by A Light Cycler480 instrument (Roche LightCycler480, Switzerland) using the SYBR Green PCR Master Mix (Roche, Switzerland), according to the manufacturer's instructions. Cycling conditions were: 95°C for 10 min, and 95°C for 15s, 55°C for 15s, and 72°C for 20s for 40 cycles. The data were normalized by the level of β‐actin expression in each individual sample. The 2^−ΔΔCt^ method was used to calculate relative expression changes. The primer sequences are listed in Table S2.

### 2.8 Western blot analysis

Western blot analysis is performed as described previously.^20^ Total protein extracted from aorta tissues and from cells by using the Total Protein Extraction Kit (AT‐022, Invent Biotechnologies, Plymouth, MN, USA) was separated by SDS‐PAGE, transferred to PVDF membrane, blocked by 5% BSA (Albumin from bovine serum) and incubated with primary antibodies overnight at 4°C. Finally, transferred blots were displayed by using chemiluminescent reagent (WBKLS0500, Millipore, Germany) and exposured by a chemiluminescence instrument (GE, Amersham Imager 600RGB).

### 2.9 Statistics analysis

All data were presented as mean ± SEM. All analyses were performed with GraphPad Prism 8 (GraphPad, San Diego, CA). Each experiment was repeated independently for a minimum of three times. Normality assumption of the data distribution was assessed using Shapiro‐Wilk test. For normally distribution, data were analysed by unpaired two‐tailed Student's *t* tests to determine the statistical difference between two groups, and one‐way ANOVA followed by Dunnett or Tukey post hoc tests were performed to determine the statistical difference between multiple groups with one variable and normally distribution. To compare multiple groups with more than one variable, two‐way ANOVA followed by Tukey or Sidak post hoc tests was used. For data with a non‐Normally distribution, we performed a nonparametric statistical analysis using the Kruskal‐Wallis test followed by the Dunn post‐hot test for multiple comparisons with one variable. In all statistical comparisons, a *p* value of <0.05 was considered statistically significant. Unless otherwise specified, each legend displays an adjusted *p* value for multiple comparisons. Non‐significant *p*‐values were not shown.

## 3 RESULTS

### 3.1 Western‐type diet increases the expression of NPRC in adipose tissue and enhances adipose inflammation in ApoE^−/−^ mice

**FIGURE 1:**
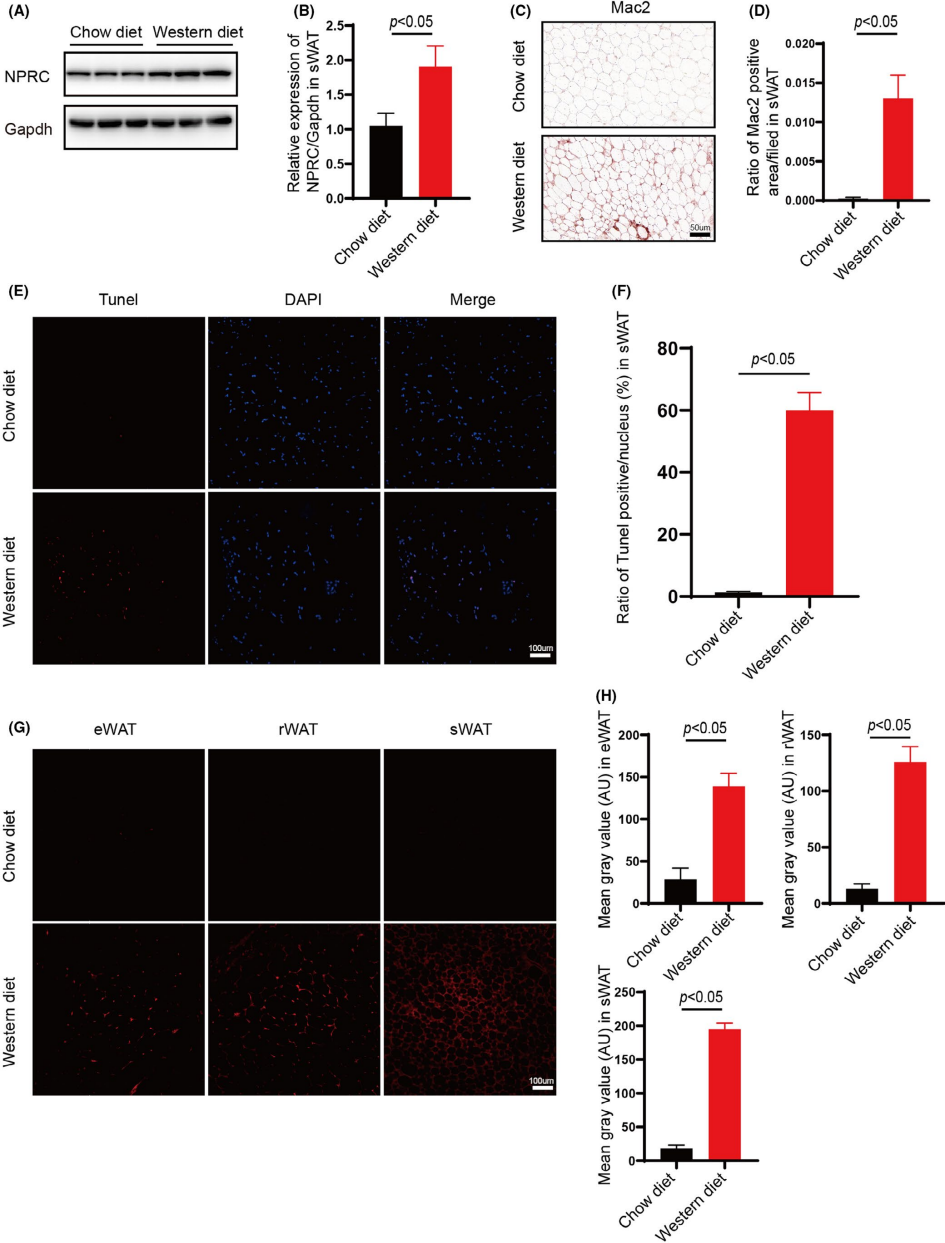
Increased NPRC expression and inflammation in white adipose tissue of western‐diet ApoE knockout hypercholesterolemic mice. (A) NPRC expression in subdermal adipose tissue of ApoE^−/−^ mice fed with chow diet and western diet measured by Western blot. (B) Quantification of relative expression in subdermal adipose tissue of NPRC in ApoE^−/−^mice fed with chow diet and western diet (*n* = 5). (C) NPRC in subdermal adipose tissue from ApoE^−/−^ mice fed with chow diet and western diet stained by immunohistochemistry. (D) Quantification of NPRC in subdermal adipose tissue from ApoE^−/−^ mice fed with chow diet and western diet stained by immunohistochemistry (*n* = 5). (E) Apoptosis assessed in subdermal adipose tissue from ApoE^−/−^ mice fed with chow diet and western diet measured by TUNEL kit (*n* = 5). WT and NPRC^−/−^ mice were measured by Western blot. (F) Quantification of apoptosis assessed in subdermal adipose tissue from ApoE^−/−^ mice fed with chow diet and western diet (*n* = 5). (G) ROS assessed in white adipose tissue from ApoE^−/−^ mice fed with chow diet and western diet measured by DHE staining. (H) Quantification of ROS assessed in white adipose tissue from ApoE^−/−^ mice fed with chow diet and western diet (*n* = 5)

To clarify the relationship among NPRC in adipose tissue, adipose inflammation and pro‐atherosclerotic diet, ApoE^−/−^ mice were fed with chow diet and western‐type diet, in which we found that a considerable enhanced expression of NPRC in white adipose tissue (Figure 1A and B), and Immunohistochemical staining showed that increased macrophage infiltration into adipose tissue of ApoE^−/−^ mice fed with western diet compared with chow diet (Figure 1C and D). Importantly, accompanied by increased expression of NPRC, we found a remarkable increase in apoptosis in white adipose tissue measured by TUNEL assay (Figure 1E and F) in mice fed with western‐type diet, which suggested that in white adipose tissue from hypercholesteremia mice, more apoptotic adipocytes needed to be cleared by macrophage infiltrated and more CLS characterized as dying or dead adipocyte surrounded by several macrophage may be viewed. Besides, reactive oxidative species (ROS) were assayed with DHE staining and we found a harmful increase in ROS in western‐type diet‐fed mice (Figure 1G and I). This suggested that high cholesterol diet induced oxidative stress in white adipocytes, which may be one of the main causes of cell apoptosis. No matter increased apoptosis or aggravated oxidative stress, more severe inflammation was induced by western‐type diet in white adipose tissue.

These data show that western‐type diet increases the expression of NPRC and inflammation in white adipose tissue of ApoE^−/−^ mice. As reported, inflammation of adipose tissue, either brown or white, may largely contribute to atherosclerosis. Therefore, we put forward a hypothesis that the gene NPRC may regulate the progress of atherosclerosis through modulating inflammation of adipose tissue.

### 3.2 Loss of NPRC enhances browning of white adipose tissue and metabolism of fat

**FIGURE 2:**
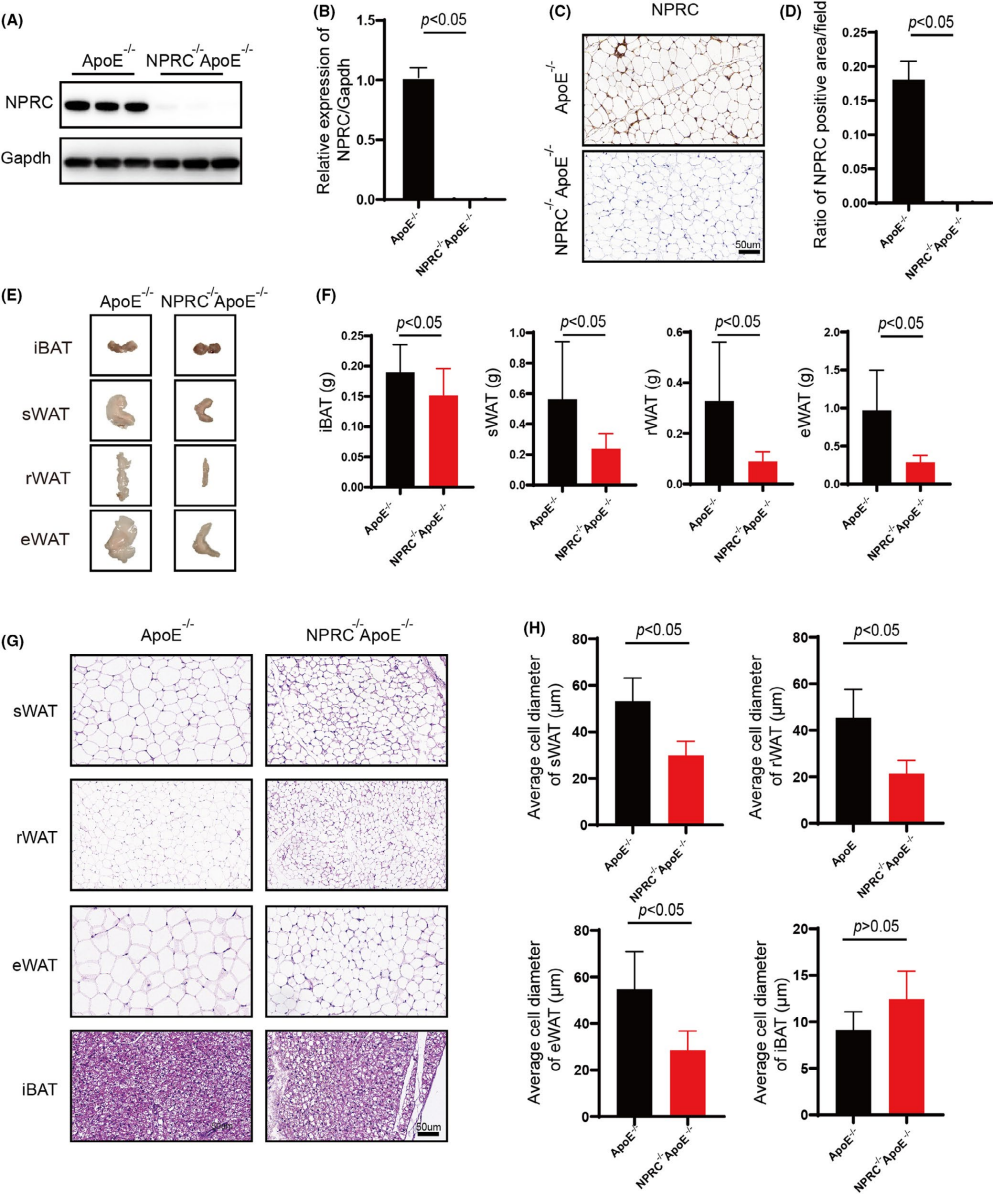
Loss of global NPRC decreases white adipose mass and white adipocyte size. (A) Expression of NPRC in subdermal adipose tissue from ApoE^−/−^NPRC^−/−^ mice and ApoE^−/−^ mice measured by Western blot. (B) Quantification of Expression of NPRC in subdermal adipose tissue from ApoE^−/−^NPRC^−/−^ mice and ApoE^−/−^ mice (*n* = 5). (C) The representative images of immunohistochemical staining for NPRC in subdermal adipose tissue in APOE^−/−^NPRC^−/−^ mice and APOE^−/−^ mice. (D) Quantification of ratio of NPRC positive area in adipose tissue (*n* = 5). (E) The representative images of adipose tissue mass of APOE^−/−^NPRC^−/−^ mice and APOE^−/−^ mice. (F) Quantification of adipose tissue mass of APOE^−/−^NPRC^−/−^ mice and APOE^−/−^ mice (*n* = 5). (G) The representative images of haematoxylin and eosin staining in subdermal white adipose tissue in APOE^−/−^NPRC^−/−^ mice and APOE^−/−^ mice. (H) Quantification of average adipocyte size in subdermal white adipose tissue (*n* = 5)

**FIGURE 3:**
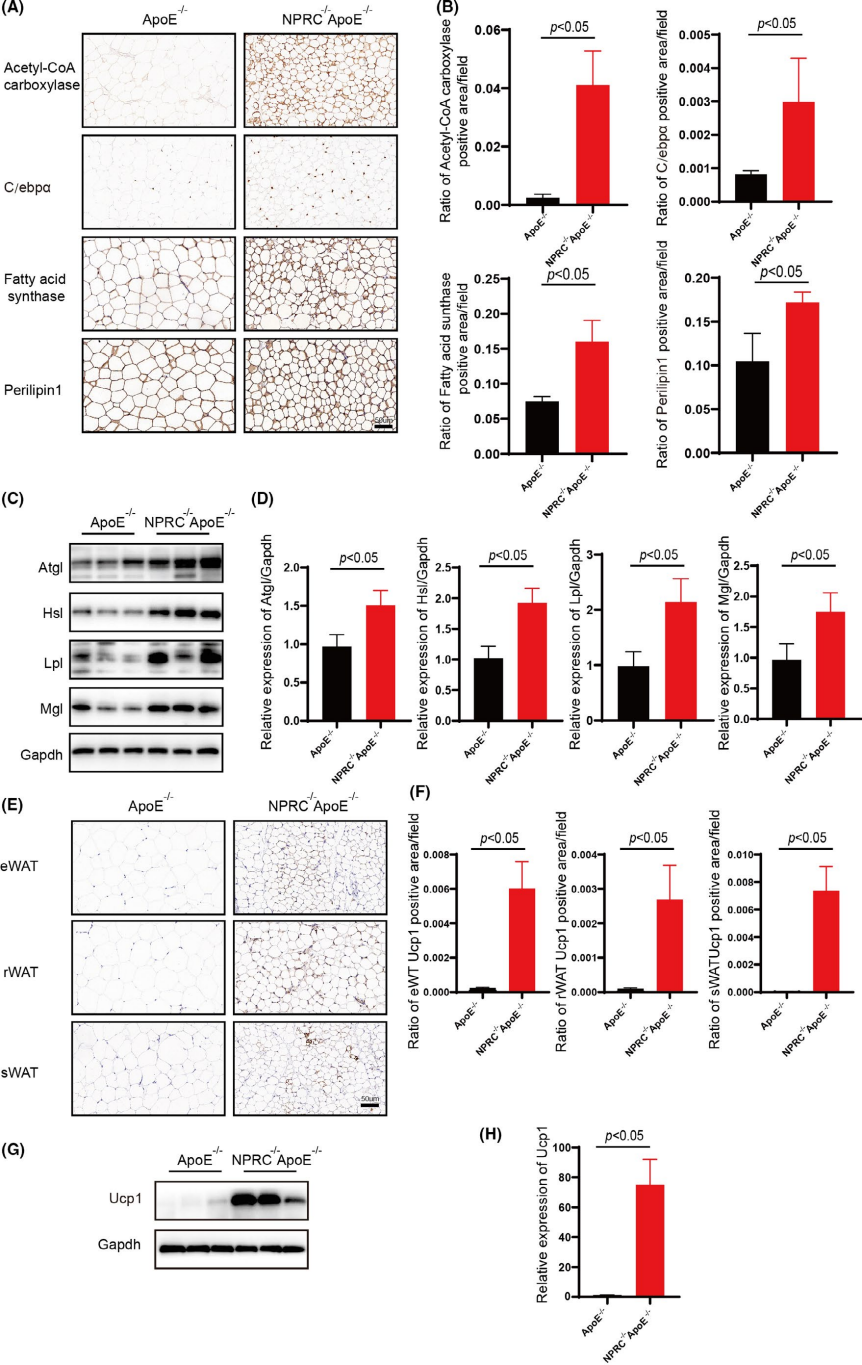
Loss of NPRC enhances browning of white adipose tissue and metabolism of fat. (A) The representative images of immunohistochemical staining for Acetyl‐CoA carboxylase, C/ebpα, fatty acid synthase, perilipin1 in subdermal white adipose tissue in APOE^−/−^NPRC^−/−^ mice and APOE^−/−^ mice. (B) Quantification of ratio of Acetyl‐CoA carboxylase, C/ebpα, fatty acid synthase, perilipin1 positive area (*n* = 5). (C) Expression of ATGL, HSL, LPL, MGL in subdermal white adipose tissue from ApoE^−/−^NPRC^−/−^ mice and ApoE^−/−^ mice measured by Western blot. (D) Quantification of ATGL, HSL, LPL and MGL in subdermal white adipose tissue from ApoE^−/−^NPRC^−/−^ mice and ApoE^−/−^ mice (*n* = 5). (E) The representative images of immunohistochemical staining for Ucp1 in subdermal white adipose tissue. (F) Quantification of ratio of Ucp1 positive area in subdermal white adipose tissue (*n* = 5). (G) Expression of Ucp1 in subdermal white adipose tissue from ApoE^−/−^NPRC^−/−^ mice and ApoE^−/−^ mice measured by Western blot. (H) Quantification of Ucp1 in subdermal white adipose tissue from ApoE^−/−^NPRC^−/−^ mice and ApoE^−/−^ mice (*n* = 5)

As mentioned above, we put forward the hypothesis that the gene NPRC may influence the macrophage infiltration and inflammation in adipose tissue mediated by western‐type diet in ApoE^−/−^ mice. We constructed NPRC^−/−^ApoE^−/−^ mice to carry on a deeper study. The Western blot and immunohistochemical assay clarified the deficiency of NPRC in adipose tissue of NPRC^−/−^ApoE^−/−^ mice (Figure 2A and B). Interestingly, we viewed a less adipose tissue volume and weight in NPRC^−/−^ApoE^−/−^ mice compared with their roommates (Figure 2E and F). Similarly, smaller white adipocytes were observed in haematoxylin and eosin‐staining tissue sections (Figure 2G and H). However, brown adipose tissue did not differ from NPRC^−/−^ApoE^−/−^ mice to ApoE^−/−^ mice. To gain insight into the underlying causes of NPRC‐deletion‐mediated change in adipose tissue, we assessed the marker of browning of adipose tissue, uncoupled protein 1 (UCP1), increases obviously, which was estimated by Western blot and immunohistochemical staining in white adipose tissue (Figure 3E‐H, Supplement Figure 1E and F). However, in brown adipose tissue, we did not view same difference. In other words, loss of NPRC induced white adipocytes browning and then regulated the metabolism of fat. Therefore, the metabolism of fat in adipose tissue from NPRC^−/−^ApoE^−/−^ mice and ApoE^−/−^ mice was assessed to find whether there was great difference, in which we observed that the synthesis markers (fatty acid synthase, acetyl‐CoA carboxylase and C/EBPα) of fat were stimulated in NPRC^−/−^ApoE^−/−^ mice measured by Western blot and immunohistochemical staining (Figure 3A and B, Supplement Figure 1A‐D). Interestingly, a similar activation of lipolysis of fat was found in NPRC^−/−^ApoE^−/−^ mice measured by Western blot of ATGL, HSL, LPL and MGL (Figure 3C and D). Among these lipolytic enzymes, ATGL was the key enzyme regulating adipose lipolysis. Deletion of ATGL induces white adipose tissue browning and increases brown adipocytes death and CLS density.^21^ In turn, more expression of ATGL not only upregulates lipolysis of fat but also decreases inflammation in adipose tissue. Besides, the markers of β‐oxidation of fat (PPAR‐γ and PGC1α) were viewed upregulation in WAT from NPRC^−/−^ApoE^−/−^ mice, which suggested that the thermogenic metabolism of fat was stimulated (Figure 6A‐D, Supplement Figure 2A–F). What is more, PPAR‐γ and PGC1α were reported to decrease the inflammation in white adipose tissue and perivascular brown adipose tissue due to their inhibition of NF‐κB signalling pathway.^22^

These results suggest that adipose tissue from NPRC^−/−^ApoE^−/−^ mice was undergoing a process of browning and active metabolism at the same time. According to the phenotype of mice, decreased fat mass was viewed in NPRC^−/−^ApoE^−/−^ mice. Therefore, despite like lipolysis, synthesis of fat was upregulated, and the net effect of loss of NPRC in adipose tissue was reduced in fat mass.

### 3.3 Loss of NPRC decreases white adipose inflammation in atherosclerotic mice

**FIGURE 4:**
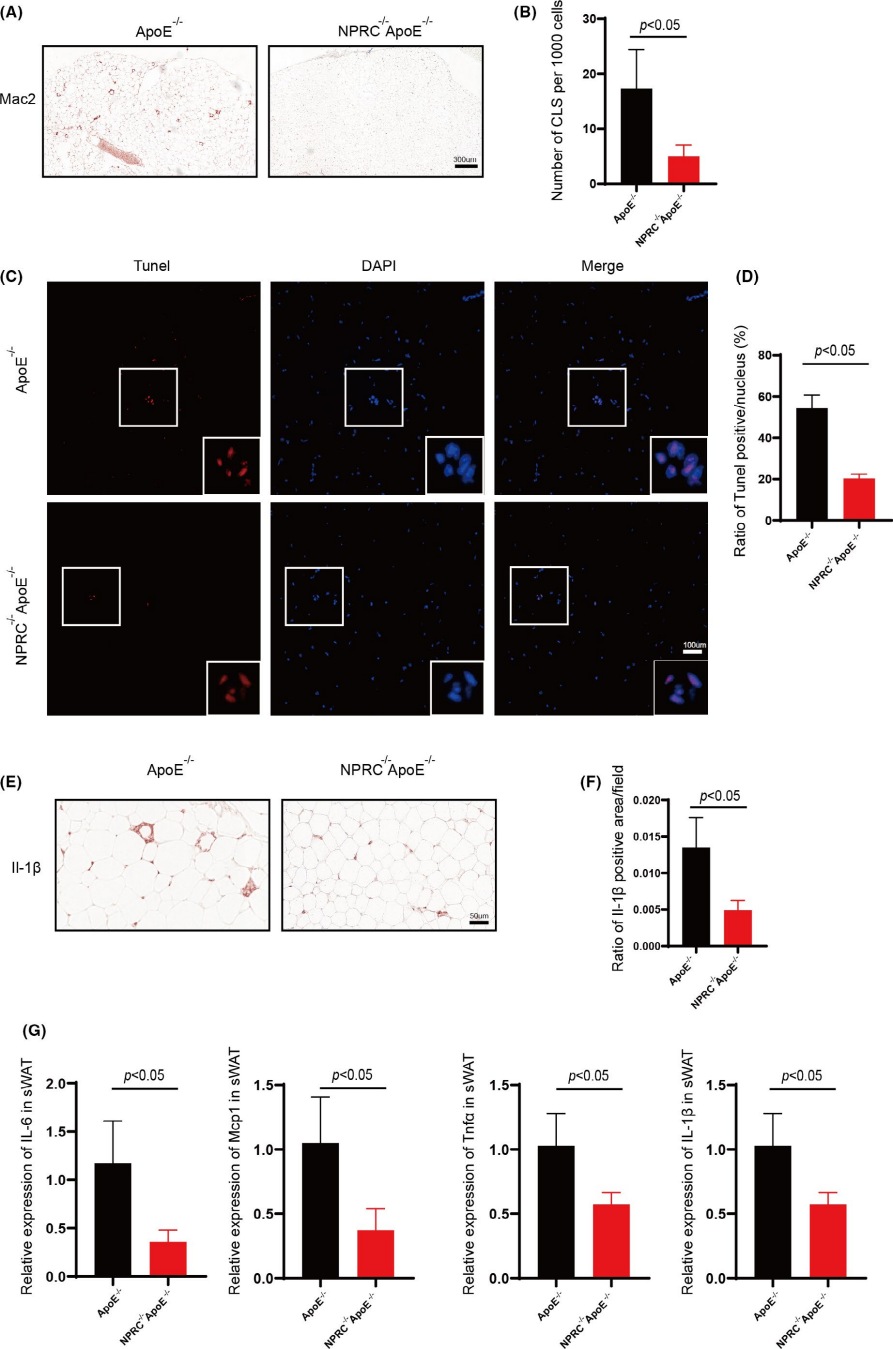
Loss of NPRC decreases white adipose inflammation and apoptosis of adipocytes in hypercholesterolemic mice. (A) The representative images of immunohistochemical staining for Mac2 in subdermal white adipose tissue. (B) Quantification of CLS density positive area (*n* = 5). (C) The representative images of TUNEL assay for subdermal white adipose tissue in APOE^−/−^NPRC^−/−^ mice and APOE^−/−^ mice. (D) Quantification of ratio of TUNEL positive pots in subdermal white adipose tissue in APOE^−/−^NPRC^−/−^ mice and APOE^−/−^ mice (*n* = 5). (E) The representative images of immunohistochemical staining for IL‐1β in subdermal white adipose tissue. (F) Quantification of ratio of IL‐1β positive area (*n* = 5). (G) IL‐6, Mcp1, TNFα and IL‐1β in ApoE^−/−^NPRC^−/−^ mice and ApoE^−/−^ mice measured by qPCR (*n* = 5)

As we expected, a pattern of decreased macrophage infiltration and less crown‐like structure in adipose tissue was observed in NPRC^−/−^ApoE^−/−^ mice compared with ApoE^−/−^ mice fed with western‐type diet (Figure 4A and B). What is more, we assessed the expression of perilipin, which is recognized as a marker of adipocyte viability, to find that white adipose tissue from NPRC^−/−^ApoE^−/−^ mice was stronger perilipin immunoreactive (Figure 3A and B, Supplement Figure 2A‐D). These data suggested that loss of NPRC may decrease the death of adipocytes.

The above data suggest that loss of NPRC decreases white adipocyte death and to be cleared by macrophage. Then, we tested the apoptosis of adipocytes in white adipose tissue by TUNEL assay to get a similar result (Figure 4C and D). Moreover, among the macrophages infiltrated into adipose tissue, a decreased expression of classic adipose tissue inflammation markers, such as IL‐1β, was viewed in NPRC^−/−^ApoE^−/−^ mice compared with ApoE^−/−^ mice (Figure 4E and F). Besides, we tested several inflammatory mediators in adipose tissue to find there was a similar reduction in expression of them such as TNFα, MCP1 and IL‐6 (Figure 4G).

These data provide evidence to prove loss of NPRC plays an important role in regulating adipose inflammation, which is engaged with reducing infiltration of inflammatory macrophages and expression of inflammatory mediators and increasing secretion of adiponectin. Loss of NPRC helps adipose tissue maintains homeostasis to reduce harmful effects to atherosclerosis.

### 3.4 Loss of NPRC decreases white adipose tissue oxidative stress and NLRP3 inflammasome activation while increases secretion of adiponectin

**FIGURE 5:**
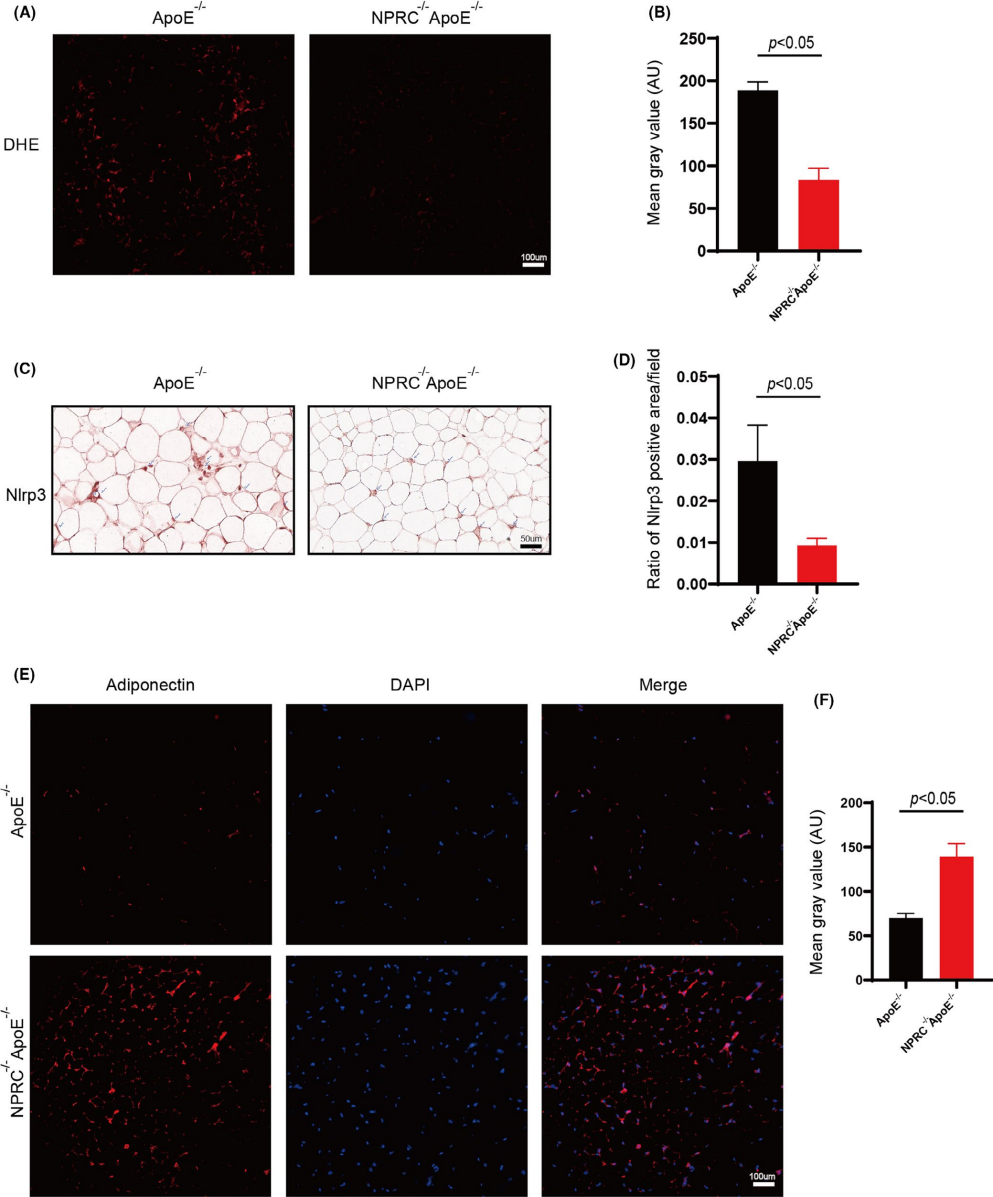
Knockdown of NPRC ameliorates oxidative stress and the expression of NLRP3 inflammasome and increases expression of adiponectin. (A) ROS assessed in subdermal adipose tissue from ApoE^−/−^ mice fed with chow diet and western diet measured by DHE staining. (B) Quantification of ROS assessed in subdermal adipose tissue from ApoE^−/−^ mice fed with chow diet and western diet (*n* = 5). (C) The representative images of immunohistochemical staining for Nlrp3 in subdermal white adipose tissue. (D) Quantification of ratio of Nlrp3 positive area (*n* = 5). (E) The representative images of immunofluorescence staining for adiponectin in subdermal white adipose tissue. (F) Quantification of mean gray value of fluorescence for adiponectin (*n* = 5)

Adipocyte death was caused by lots of factors, among which oxidative stress induced by large amounts of reactive oxygen species from ER stress or mitochondrial dysfunction.^23^ Therefore, ROS were measured in adipose tissue to find an obvious decrease in NPRC^−/−^ApoE^−/−^ mice, which suggested that loss of NPRC may alleviate oxidative stress induced by western‐type diet (Figure 5A and B). Furthermore, CLS density is usually directly proportional to increased expression of inflammatory marker genes, as previously described.^10^, ^24^ Among these inflammatory marker genes, NLRP3 inflammasome was recognized as an important factor in inflammation. As expected, reduction in the expression of NLRP3 inflammasome and IL‐1β in WAT from NPRC^−/−^ApoE^−/−^ mice were viewed in white adipose tissue, which suggested that downregulation of brown fat inflammation and CLS formation in white adipose tissue might credit to the inhibition of inflammasome activation (Figure 5C and D). Moreover, adiponectin, as an anti‐inflammatory adipocytokine secreted by adipocytes, was measured by immunofluorescence in white adipose tissue to find a significant stronger expression in NPRC^−/−^ApoE^−/−^ mice (Figure 5E and F). As reported, more adiponectin in serum exerted an anti‐atherosclerotic effect on plaque.

### 3.5 Loss of NPRC activates cAMP/PKA signalling pathway

**FIGURE 6:**
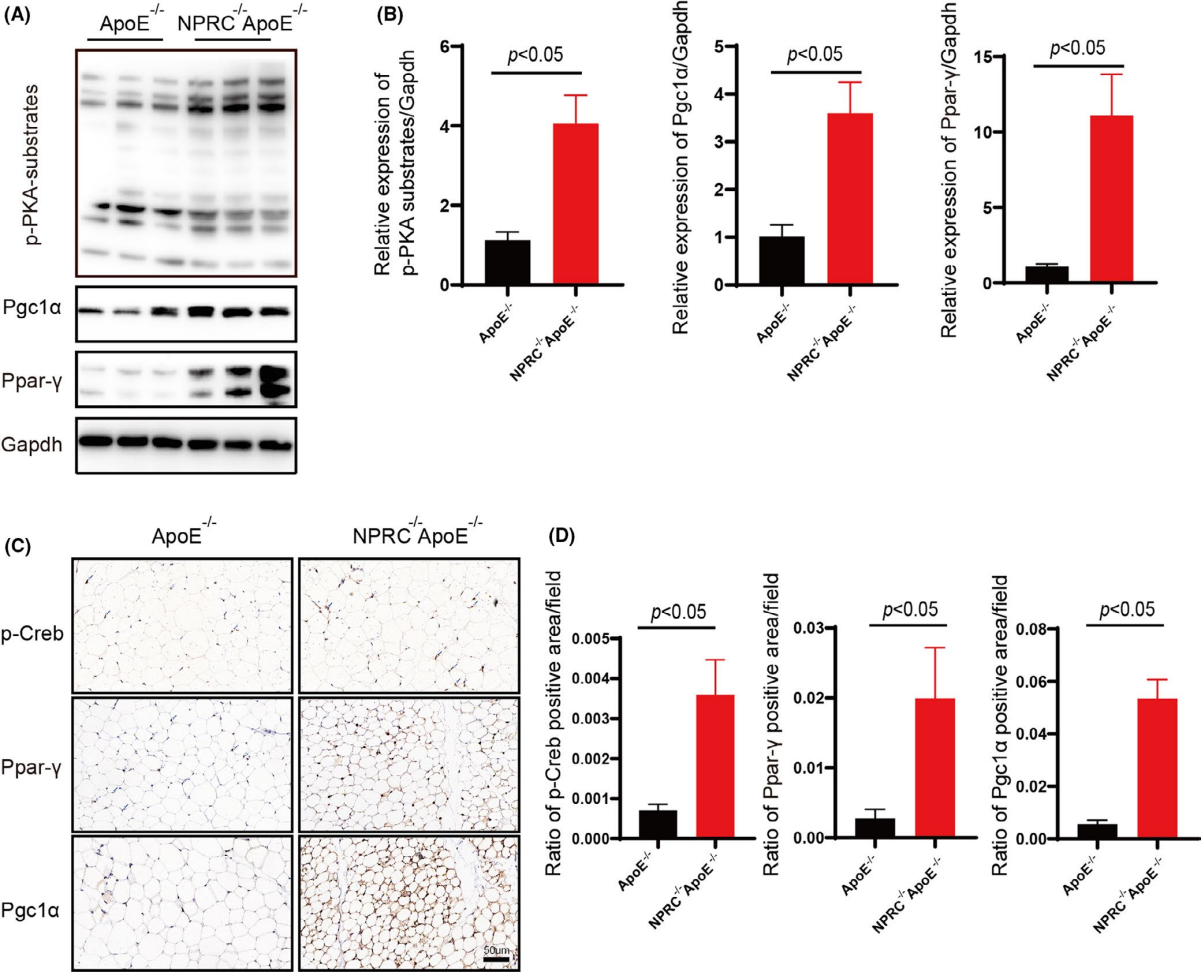
Loss of NPRC activates cAMP/PKA signalling pathway. (A) Expression of p‐PKA‐substrate, PPAR‐γ, PGC1α in subdermal white adipose tissue from APOE^−/−^NPRC^−/−^ mice and APOE^−/−^ mice. (B) Quantification of expression of p‐PKA‐substrate, PPAR‐γ and PGC1α in subdermal white adipose tissue from ApoE^−/−^NPRC^−/−^ mice and ApoE^−/−^ mice (*n* = 5). (C) The representative images of immunofluorescence staining for p‐Creb, PPAR‐γ and PGC1α in subdermal white adipose tissue from ApoE^−/−^NPRC^−/−^ mice and ApoE^−/−^ mice. (D) Quantification of ratio of p‐Creb, PPAR‐γ and PGC1α positive area (*n* = 5)

As elaborated above, to further study the mechanisms of increased browning and active state of adipose tissue in NPRC^−/−^ApoE^−/−^ mice and the relationship between these changes and adipose inflammation, we tested cAMP/PKA signalling pathway according to the knowledge of natriuretic peptide family described before. We measured p‐PKA substrates (Figure 6A and B) and the downstream p‐CREB by Western blot and immunohistochemistry staining (Figure 6A‐D, Supplement Figure 2A‐F). We found PKA signalling pathway was activated obviously and the expression of effective proteins of PKA pathway also was viewed considerable upregulation in WAT from NPRC^−/−^ApoE^−/−^ mice. As reported, PKA signalling pathway activated the phosphorylation of p38 and then induced the brown fat thermogenic programme in mouse and human adipocytes,^25^ which was similar with our data. Moreover, PKA, as a recognized anti‐inflammatory factor, can play its role in inflammation of sorts of disease through multiple ways.

## 4 DISCUSSION

Under healthy conditions, WAT acts as a lipid sink by storing lipids, which protects from atherosclerosis through preventing lipids to accumulate in the circulation.^26^ However, during obesity, WAT is characterized by chronic local inflammation and the presence of crown‐like structures, where aggregates of macrophages surround dead adipocytes to clear the extracellular space of adipocyte debris.^24^, ^27^, ^28^ In our study, we found that ApoE^−/−^ mice fed with western‐type diet not only induced atherosclerosis but also stimulated adipose inflammation characterized by increased CLS formation, which suggested inflammation of WAT may accompany with and aggravate the process of atherosclerosis. Therefore, we come up with an idea that decreased inflammation of WAT in atherosclerotic mice may be beneficial to the treatment of atherosclerosis. As mentioned above, natriuretic peptide family play an important role in adipose metabolism. Interestingly, our data showed that NPRC, the clearance receptor of ANP, BNP and CNP, exerted an obvious effect on regulating inflammation of WAT induced by high cholesterol diet in ApoE^−/−^ mice.

It was reported that the number of pro‐inflammatory M1 type macrophages, cytotoxic T cells, TH1 cells, B cells and mast cells increases, whereas the anti‐inflammatory M2 type macrophages, regulatory T cells decrease in inflammatory WAT.^29^ Our data showed that deletion of NPRC alleviated adipocytes death and decreased CLS density in WAT from ApoE^−/−^ mice fed with western‐type diet. Furthermore, deletion of NPRC reduced the infiltration of macrophages, which may credit to less secretion of inflammatory mediators and chemokines, such as TNFα, MCP1 and IL‐6 from hypertrophic adipocytes. As reported, adipocyte hypertrophy is associated with several pro‐inflammatory signalling pathway, such as JNK and NF‐κB pathways.^30^ In NPRC^−/−^ApoE^−/−^ mice, browning of WAT obviously was viewed, which induced a comparable reduction in the size of adipocytes. Besides, oxidative stress, ER stress and increased release of pro‐inflammatory adipokines and chemokines also contribute to adipose inflammation.^30^, ^31^ Our results suggested that deletion of NPRC decreased reactive oxidative species in WAT, which may help maintain the mitochondrial function and adipocytes viability.

What is more, many types of adipokines released by WAT exert direct immunomodulatory effects on the vascular wall. Among these adipokines, adiponectin and omentin have a protective role in the process of atherosclerosis,^32^ while leptin was shown to promote atherosclerosis development.^33^ Our data showed an obvious increase in the expression and secretion of adiponectin in WAT from NPRC^−/−^ApoE^−/−^ mice compared with ApoE^−/−^ mice. According to the studies of Yamauchi and Kadowaki, increasing the levels of adiponectin is a promising approach to reduce adipose inflammation and may contribute to alleviate atherosclerosis development.^34^ In vitro, adiponectin induces NO production in human aortic endothelial cells via activation of endothelial NO synthase activity.^35^ Besides, adiponectin has been demonstrated to inhibit TNFα and IL‐8‐induced ICAM1, VCAM1 and E‐selectin.^36^ These results suggested that deletion of NPRC may play a beneficial role in alleviating atherosclerosis by the increased secretion of adiponectin.

Given the above results showed the WAT inflammation decreased in NPRC^−/−^ApoE^−/−^ mice fed with western‐type diet, we set further research on the underlying mechanism. As reported, we viewed an obvious browning of white adipose tissue in NPRC^−/−^ApoE^−/−^ mice. Interestingly, brown adipose tissue from two groups differed little. Importantly, data from mice stress that BAT ablation leads to obesity^37^ and whitening of BAT caused by ATGL‐deletion induces BAT inflammation.^21^ What is more, browning of subcutaneous adipose tissue is reported to be inhibited by adipocyte ceramides but the inflammation of subcutaneous adipose tissue is found to be more serious.^38^ On the contrary, BAT transplantation attenuated the obesity‐associated adipose tissue inflammation in terms of decreased pro‐inflammatory M1‐macrophages, cytotoxic T cells and restored anti‐inflammatory regulatory T cells (Tregs) in the eWAT.^39^ Besides, increased browning of WAT caused by Kynurenic acid and Gpr35 is found to regulate inflammation of adipose tissue.^40^ Therefore, browning of WAT viewed in NPRC^−/−^ApoE^−/−^ mice may contribute to the decreased adipose inflammation. The process of browning is characterized by the upregulation of expression of UCP1, which was thought to be induced by activated cAMP‐PKA signalling pathway mediated by deletion of NPRC. Besides, PPAR‐γ and PGC1α are important target genes of PKA.^41^, ^42^ In our studies, expression of PPAR‐γ and PGC1α was stimulated by deletion of NPRC, which may be mediated by activated PKA signalling pathway as well. It was reported that the promotor of the adiponectin gene contained a functional PPRE that was activated by PPAR‐γ.^43^ At the same time, adiponectin induced by PPAR‐γ maintains extracellular space of adipocytes to keep adipocytes metabolically active during the expansion of adipocytes.^44^ Furthermore, activated PPAR‐γ exerts an anti‐inflammatory effect by suppressing the NLRP3 inflammasome in several disease.^45^, ^46^, ^47^ Therefore, we tested the expression of NLRP3 inflammasome in WAT from NPRC^−/−^ApoE^−/−^ mice and ApoE^−/−^ mice and found deletion of NPRC indeed decreased the expression of NLRP3 inflammasome and its downstream effector, IL‐1β.

NPRC is recognized as a clearance receptor for ANP, BNP and CNP. In our study, knockout of global NPRC may induce upregulated natriuretic peptides due to the defective clearance of them by NPRC. Besides, the increased natriuretic peptides in serum may exert effects on adipose tissue because of the expression of NPRA and NPRB in adipose tissue. It is reported that ANP can be able to prevent PCSK9 overexpression and control LDLR through its receptor NPRA.^48^ Besides, decreased ANP levels resulted from cardiac autophagy deficiency disrupts myocardial‐adipose cross talk, which leads to increased fat accumulation.^49^ What is more, CNP produced by the endothelial cells can regulate white adipose hypertrophy and insulin resistance during high‐fat diet‐induced obesity.^50^ Therefore, natriuretic peptides may play roles in the inflammation of adipose tissue induced by pro‐atherosclerotic diet, and more studies are needed to explore the effects of natriuretic peptides on inflammation of adipose tissue.

Taken together, in atherosclerotic patients, inflammation of adipose tissue is a common complication that should not be ignored, which may influence largely the process and treatment of atherosclerosis. Our findings suggest that aiming at decreasing the fat inflammation may provide beneficial effects to treat atherosclerosis.

## CONFLICT OF INTEREST

The authors declare that they do not have any conflict of interest that could affect the job done in this paper.

## AUTHOR CONTRIBUTIONS

**Cheng Cheng:** Conceptualization (lead); Data curation (lead); Formal analysis (lead); Investigation (lead); Writing‐original draft (lead). **Fei Xue:** Conceptualization (supporting). **Wenhai Sui:** Conceptualization (supporting). **Linlin Meng:** Conceptualization (supporting). **Lin Xie:** Conceptualization (supporting). **Cheng Zhang:** Methodology (lead). **Jianmin Yang:** Writing‐original draft (lead). **Yun Zhang:** Funding acquisition (lead).

## Supporting information

Fig S1

Fig S2

Appendix S1

## Data Availability

The data that support the findings of this study are available on request from the corresponding author. The data are not publicly available due to privacy or ethical restrictions.
