# Supplementary figures and images for: Deletion of natriuretic peptide receptor C alleviates adipose tissue inflammation in hypercholesterolemic Apolipoprotein E knockout mice
Source: J Cell Mol Med. 2021 Sep 15;25(20):9837–50. doi: 10.1111/jcmm.16931 (PMC8505842; doi:10.1111/jcmm.16931)

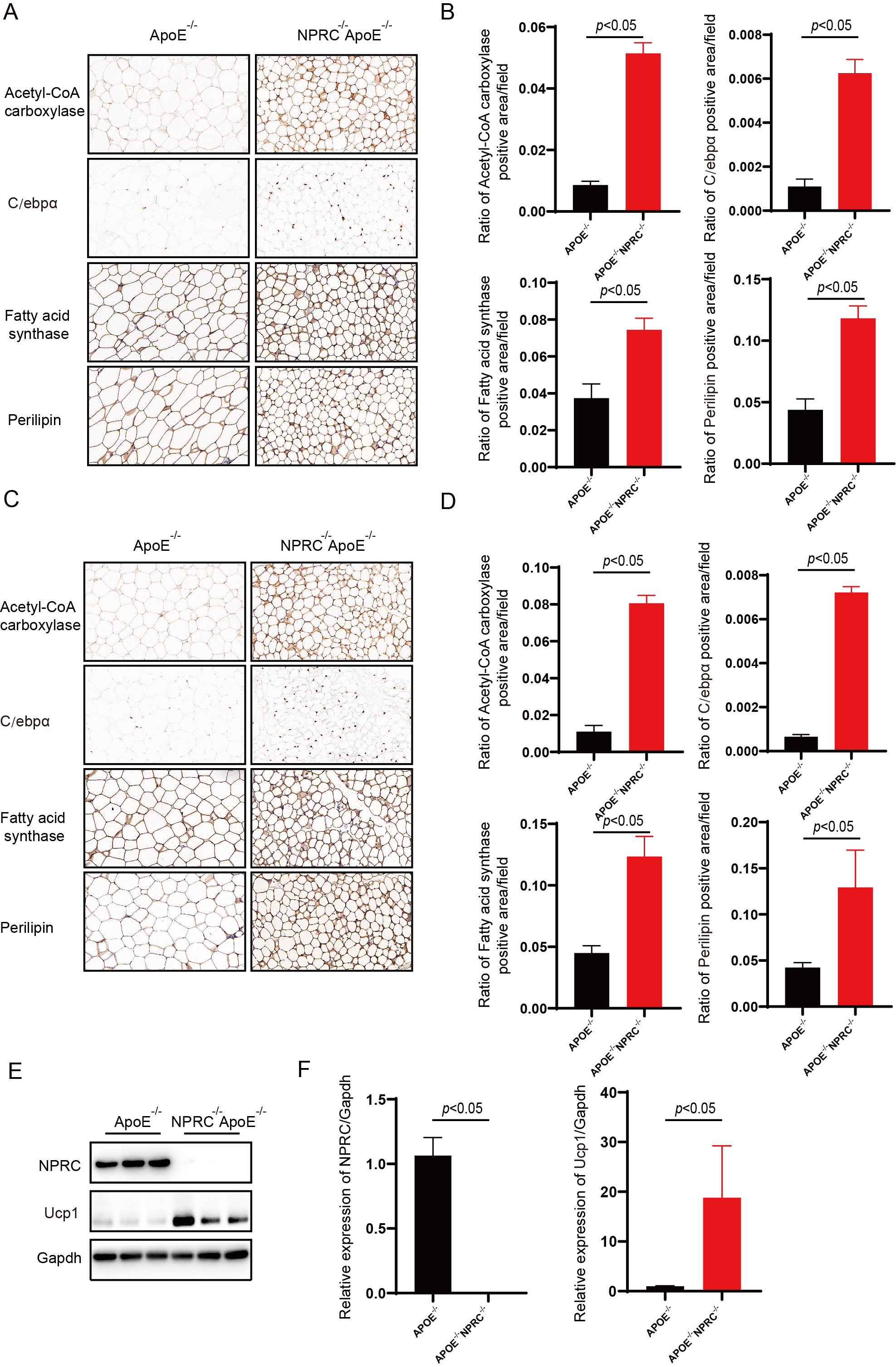

Supplement: Supplementary file 1 — Fig S1 [file JCMM-25-9837-s003.jpg]

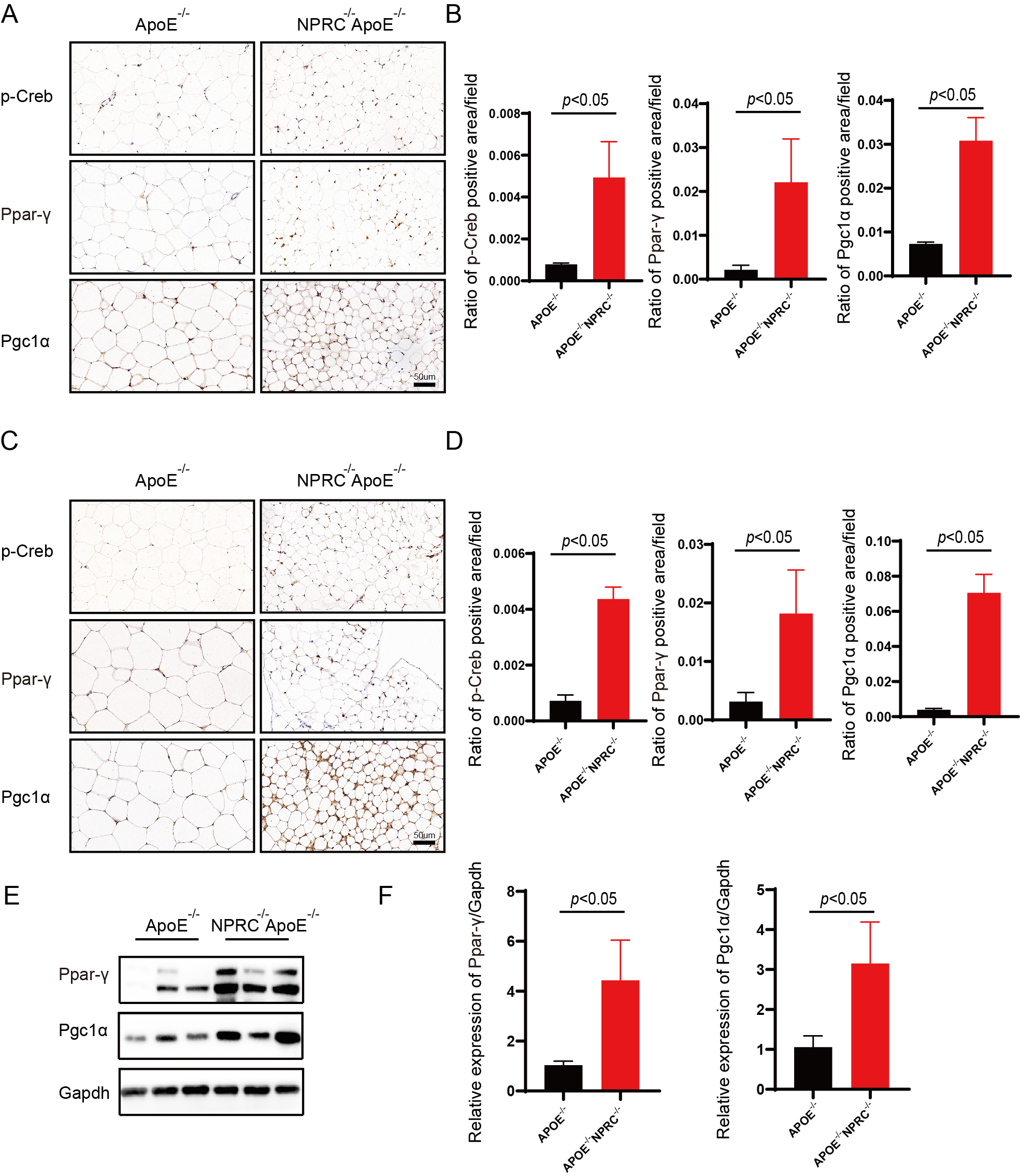

Supplement: Supplementary file 2 — Fig S2 [file JCMM-25-9837-s001.jpg]
