# Supplementary material for: Deletion of natriuretic peptide receptor C alleviates adipose tissue inflammation in hypercholesterolemic Apolipoprotein E knockout mice
Source: J Cell Mol Med. 2021 Sep 15;25(20):9837–50. doi: 10.1111/jcmm.16931 (PMC8505842; doi:10.1111/jcmm.16931)
Supplement: Supplementary file 3 — Appendix S1 [file JCMM-25-9837-s002.docx]

**Supplementary Table 1. Antibodies and manufacturers**

| Antibody | Company | Catalog No. | Concentrations |
| --- | --- | --- | --- |
| NPRC | Sigma-Aldrich | AV34717 | IHC: 1:100, Western blot: 1:1000 |
| GAPDH | Cell Signaling Technology | #2118 | Western blot: 1:1000 |
| IL-1β | Abcam | ab9722 | IHC: 1:200 |
| p-Creb | Cell Signaling Technology | #9198 | Western blot: 1:1000 |
| Pparγ | Cell Signaling Technology | #2435 | Western blot: 1:1000 |
| Pgc1α | Cell Signaling Technology | #2178 | Western blot: 1:1000 |
| PKA-substrate | Cell Signaling Technology | #9624 | Western blot: 1:1000 |
| Mac2 | Abcam | Ab76466 | IHC: 1:200 |
| Nlrp3 | Abcam | Ab263899 | IHC: 1:200 |
| Acetyl-CoA carboxylase | Cell Signaling Technology | #3676 | IHC: 1:200 |
| C/ebpα | Cell Signaling Technology | #8178 | IHC: 1:200 |
| Fatty acid synthase | Cell Signaling Technology | #3180 | IHC: 1:200 |
| perilipin1 | Cell Signaling Technology | #9349 | IHC: 1:200 |
| ATGL | Abcam | Ab109251 | Western blot: 1:1000 |
| HSL | Abcam | Ab45422 | Western blot: 1:1000 |
| LPL | Abcam | Ab91606 | Western blot: 1:1000 |
| MGL | Abcam | Ab77398 | Western blot: 1:1000 |
| Ucp1 | Abcam | Ab234430 | Western blot: 1:1000 |
| Adiponectin | Abcam | Ab22554 | IHC: 1:200, IF: 1:100 |

**Supplementary Table 2. Prime sequence of genes**

| **Gene name** | **Sequence 5’-3’** |
| --- | --- |
| mouse *β-actin* forward | CCACACCCGCCACCAGTTCG |
| mouse *β-actin* reverse | TACAGCCCGGGGAGCATCGT |
| mouse *tnf-α* forward | CCCTCACACTCAGATCATCTTCT |
| mouse *tnf-α r*everse | GCTACGACGTGGGCTACAG |
| mouse *il-6* forward | AGTTGCCTTCTTGGGACTGA |
| mouse *il-6* reverse | TCCACGATTTCCCAGAGAAC |
| mouse *il1β* forward | ACCTTCCAGGATGAGGACATGA |
| mouse *il1β* reverse | AACGTCACACACCAGCAGGTTA |

**Supplement Figure 1.** Loss of NPRC enhances browning of epididymis and perirenal white adipose tissue and metabolism of fat.

A, The representative images of immunohistochemical staining for Acetyl-CoA carboxylase, C/ebpα, Fatty acid synthase, perilipin1 in epididymis white adipose tissue in APOE^-/-^NPRC^-/-^ mice and APOE^-/-^ mice.

B, Quantification of ratio of Acetyl-CoA carboxylase, C/ebpα, Fatty acid synthase, perilipin1 positive area (n=5).

C**,** The representative images of immunohistochemical staining for Acetyl-CoA carboxylase, C/ebpα, Fatty acid synthase, perilipin1 in perirenal white adipose tissue in APOE^-/-^NPRC^-/-^ mice and APOE^-/-^ mice.

D**,** Quantification of ratio of Acetyl-CoA carboxylase, C/ebpα, Fatty acid synthase, perilipin1 positive area (n=5).

E**,** Expression of NPRC and Ucp1 in epididymis white adipose tissue from APOE^-/-^NPRC^-/-^ mice and APOE^-/-^ mice.

F**,** Quantification of expression of NPRC and Ucp1 in epididymis white adipose tissue from APOE^-/-^NPRC^-/-^ mice and APOE^-/-^ mice (n=5).

**Supplement Figure 2.** Loss of NPRC activates cAMP/PKA signalling pathway in epididymis and perirenal white adipose tissue.

A, The representative images of immunohistochemical staining for p-Creb, PPAR-γ, PGC1α in epididymis white adipose tissue in APOE^-/-^NPRC^-/-^ mice and APOE^-/-^ mice.

B, Quantification of ratio of p-Creb, PPAR-γ, PGC1α positive area (n=5).

C**,** The representative images of immunohistochemical staining for p-Creb, PPAR-γ, PGC1α in perirenal white adipose tissue in APOE^-/-^NPRC^-/-^ mice and APOE^-/-^ mice.

D**,** Quantification of ratio of p-Creb, PPAR-γ, PGC1α positive area (n=5).

E**,** Expression of PPAR-γ, PGC1α in epididymis white adipose tissue from APOE^-/-^NPRC^-/-^ mice and APOE^-/-^ mice.

F**,** Quantification of expression of PPAR-γ, PGC1α in epididymis white adipose tissue from APOE^-/-^NPRC^-/-^ mice and APOE^-/-^ mice (n=5).
